# In Silico Analysis of Common Long Noncoding RNAs in *Schistosoma mansoni* and *Schistosoma haematobium*

**DOI:** 10.1155/2021/6617118

**Published:** 2021-02-15

**Authors:** Serhat Sirekbasan, Tugba Gurkok Tan

**Affiliations:** Department of Medical Laboratory Techniques, Eldivan Vocational School of Health Services, Çankırı Karatekin University, Çankırı, Turkey

## Abstract

**Background:**

Schistosomiasis caused by *Schistosoma* parasites is one of the most common parasitic infections worldwide. Genetic regulation of the genus *Schistosoma*, which has different developmental stages throughout its life, is quite complex. In these parasites, thousands of long noncoding RNAs (lncRNAs) estimated to be functional were identified. Identifying the transcripts expressed in common and detecting their functions for better understanding of the role of these lncRNAs require a comparative study.

**Methods:**

Assembled RNA-seq datasets belonging to *S. mansoni* and *S. haematobium* were obtained from the National Center for Biotechnology. A basic local alignment search tool (BLASTN) analysis was conducted against previously constructed lncRNA library to identify the common lncRNAs between two species. LncRNAs target genes and their gene ontology annotation was performed.

**Results:**

In *S. mansoni* and *S. haematobium*, 5132 and 3589 lncRNA transcripts were detected, respectively. These two species had 694 lncRNAs in common. A significant number of lncRNAs was determined to be transcribed from sex chromosomes. The frequently expressed lncRNAs appear to be involved in metabolic and biological regulation processes.

**Conclusions:**

These two species share similar lncRNAs; thus, this finding is a clue that they might have similar functions. In sexual development, they especially might play important roles. Our results will provide important clues to further studies about interactions between human hosts and parasites and the infection mechanisms of *Schistosoma* parasites.

## 1. Introduction

Schistosomiasis caused by Platyhelminthes of the genus *Schistosoma* is one of the most serious parasitic diseases in humans. It affects more than 240 million people in 78 countries worldwide and causes approximately 200,000 deaths per year [1]. Three main *Schistosoma* species can infect humans: (1) *S. haematobium*, (2) *S. mansoni*, and (3) *S. japonicum*. *S. mansoni* and *S. japonicum* cause intestinal schistosomiasis, whereas *S. haematobium* causes urogenital schistosomiasis [2, 3].

In contrast to other trematodes, the *Schistosoma* genus shows gender differentiation (male and female) [4], which have eight pairs of chromosomes, seven of which are autosomes and one is a pair of sex chromosomes [5]. In the life cycle of the *Schistosoma* species, intermediate and definite hosts with different developmental stages are found; hence, complex morphological changes in these organisms occur [6].

The elucidation of all these host-parasite relationships depends on increasing our knowledge of the *Schistosoma* genome, transcriptome, and proteome. The genetic changes or similarities that shape the evolution of the parasite are likely to play a role in host infectivity, development and differentiation, drug susceptibility, pathogenicity, and immunogenicity [6].

With the evolution of animal multicellularity, several regulatory gene expression systems developed, such as transcription factors and noncoding RNAs. Therefore, it is important to explain the regulation of gene expression in these organisms in addition to the many environmental factors required for the formation of schistosomiasis. Formerly, the only well-known ncRNAs were the ribosomal RNAs (rRNA), transfer RNAs (tRNA), and small RNAs. Nevertheless, with the advancement of high-throughput sequencing techniques and computational pipelines, new ncRNAs and their functions in genome and gene regulation has been elucidated. One of the newly characterized ncRNAs is the long noncoding RNAs (lncRNAs) lncRNAs. LncRNAs are defined as noncoding transcripts longer than 200 nucleotides and do not encode an open reading frame (ORF) of more than 100 amino acids [7]. They have important roles in gene expression, developmental and differentiation processes, and genomic imprinting [8, 9]. Besides, lncRNAs interact with other RNA species, such as microRNAs and mRNAs [10].

Classification of lncRNAs is based on several characters, such as their genomic localizations, transcript length, association with annotated protein-coding genes, and/or sequence and structure conservation [11, 12]. LncRNAs can be placed into several categories: (1) long intergenic noncoding RNAs (lincRNAs), (2) intronic lncRNAs (transcribed within the introns of protein-coding genes), or (3) antisense lncRNAs [8]. It has been reported that despite most protein-coding sequences, lncRNAs rapidly evolve and display poor primary sequence similarity between species [13].

Multicellular organisms include thousands of different lncRNA sequences in their genomes [14]. To date, from protozoans to mammals, a huge number of lncRNAs have been identified [15–18]. Over the past several decades, a number of studies on lncRNAs in *Schistosoma* species have been conducted [19, 20]. However, molecular studies of *Schistosoma* species ncRNAs are limited.

In this study, we aimed to determine the homology-based putative protected lncRNA sequences in *S. mansoni* and *S. haematobium* species and to determine their common functions among species according to their target genes.

## 2. Materials and Methods

### 2.1. Data Sources

RNA-seq datasets were obtained from Transcriptome Shotgun Assembly (TSA) database from National Center for Biotechnology Information (NCBI) under accession numbers GDUI01 (*S. mansoni*) and GGGJ01 (*S. haematobium*).

### 2.2. Homology-Based Identification of lncRNAs

Sequence homology between *S. mansoni* and *S. haematobium* lncRNAs was analyzed using a basic local alignment search tool (BLASTN) [21]. Both *S. mansoni* and *S. haematobium* assembled reads were aligned with the *S. mansoni* lncRNA library [20]. The sequences with >90% identity and a *p* value of 10^−5^ were predicted to be candidate lncRNAs.

### 2.3. Selecting the Transcripts according to Their Coding Potential

After finding the candidate lncRNA encoding transcripts, we filtered <200 bp, and the remaining transcripts were uploaded to the coding potential calculator (CPC) website (http://cpc.cbi.pku.edu.cn) for coding potential analysis [22]. CPC provides coding probability, isoelectric points, and Fickett scores and contributes a probability as to whether the transcripts are coding or noncoding. Parameters for the website were set to use only the forward strand. All transcripts with CPC scores >0.5 were discarded and remaining transcripts were considered as noncoding. Also, candidates were then searched against Pfam protein database (http://pfam.xfam.org/). Noncoding transcripts were used for further analysis.

### 2.4. Putative Target Gene Prediction and Enrichment Analysis

Previously reported antisense targets of the lncRNAs [20] were detected for the transcripts detected in our experiments. Target genes gene ontology (GO) annotation, including the GO biological process (BP), cellular component (CC), and molecular function (MF), was conducted using Uniprot database [23]. Also, the classification of the lncRNAs was performed according to a previous study [20].

## 3. Results

### 3.1. Transcriptome-Wide Identification of LncRNAs

In order to explore the lncRNAs, we used egg, adult male, and adult female assembled transcriptome libraries of *S. mansoni* and *S. haematobium*. Several steps of the bioinformatics pipeline were used to detect lncRNAs. Transcripts (23,678 and 31,591 transcripts) were previously created in *S. mansoni* [24] and *S. haematobium* (unpublished data), respectively.

**Figure 1:**
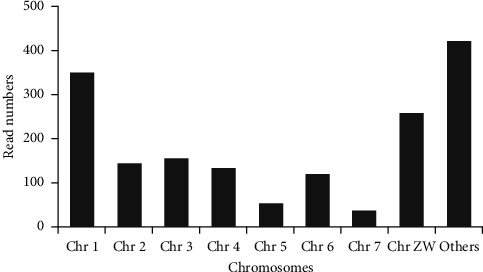
Location of the long noncoding RNA (lncRNA) transcripts and their read numbers in *S. mansoni* chromosomes. Others, lncRNAs that have not been mapped to any chromosomes.

We performed BLASTN to detect the known lncRNAS in *S. mansoni* and found 28355 hits. After removing the transcripts that were <200 bp, the number of remaining transcripts was 6110. The transcripts greater than 200 bp were also run through CPC. Of the 6110 sequences analyzed by CPC, 5132 were classified as noncoding. Among 5132 lncRNA transcripts found to be noncoding, we detected 1669 that were unique. Using the previous data created by Vasconcelos et al. [20], our results demonstrated that 213 of them had antisense lncRNAs, whereas 1456 of them were shown to be lincRNAs. We also detected the count numbers of *S. mansoni* lncRNAs in the RNA-seq library. SmAS00326 (27 read numbers) and SmLINC01644 (24 read numbers) were the most abundant lncRNAs (Table 1). The distribution of the lncRNAs to chromosomes showed that most of the lncRNA encoding genes were located at chromosome 1 and chromosome ZW (Figure 1).

In the results of *S. haematobium*, we detected 4372 transcripts longer than 200 bp from which a total of 3589 were represented as noncoding based on CPC analysis. We also detected 509 lncRNA encoding transcripts that were capable of targeting several *S. mansoni* mRNAs. All of these belonged to the antisense lncRNAs. The majority of *S. haematobium* lncRNAs were found to be intergenic (82%). Analysis of the abundance of lncRNAs revealed that SmLINC01683 was the most abundant with 27 transcripts (Table 1). Our results revealed that the number of lncRNA transcripts belonging to *S. mansoni* was higher than *S. haematobium*.

**Figure 2:**
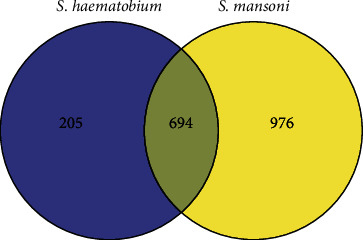
Venn diagram demonstrating shared and unique lncRNA transcripts.

The number of common lncRNAs between *S. haematobium* and *S. mansoni* species was 694 (Figure 2). The most abundant type was lincRNAs with the rate of 85%. A large number of their target annotations were found to be uncharacterized. SmAS00186 and SmAS00326 were represented by the highest number of common transcripts.

### 3.2. Target GO Enrichment Analyses

To identify and annotate the GO categories of the target genes in both *S. mansoni* and *S. haematobium*, we conducted GO enrichment analyses in terms of biological processes, molecular functions, and cellular components. The identification was carried out using UniProt.

**Figure 3:**
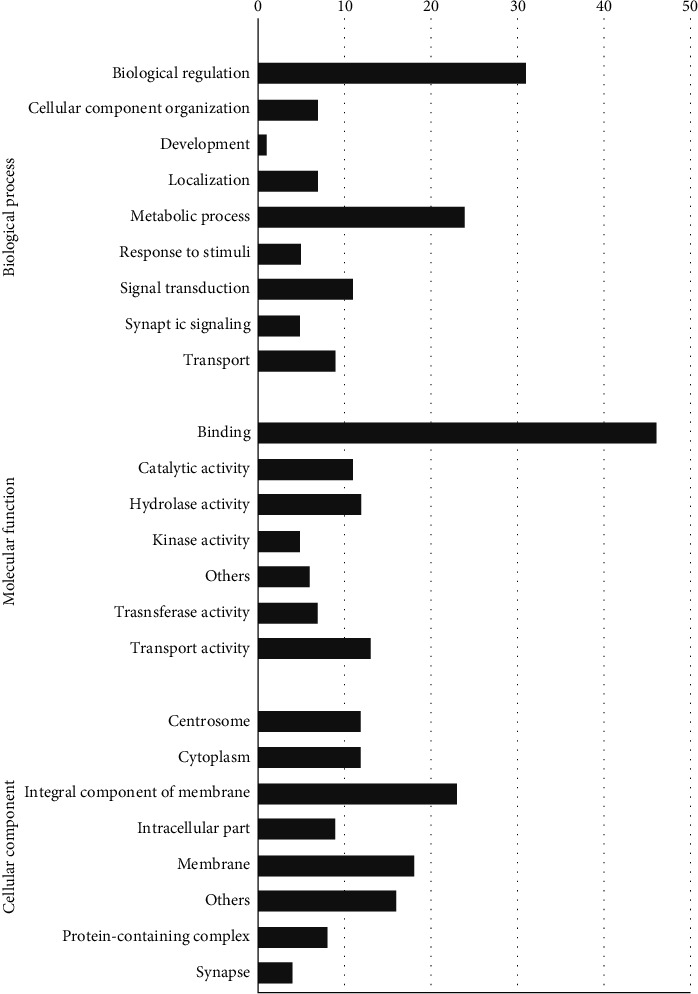
Percentage representation of gene ontology (GO) enrichment analysis of lncRNA targets in *S. mansoni*.

In *S. mansoni*, only 10% of the transcripts had target genes. In biological processes, the majority of the target genes existed in relation to biological regulation (31%) and metabolic processes (24%) as shown in Figure 3. The GO analysis revealed binding of 46% in relation to molecular function and 23% in relation to integral membrane components in cellular compartments.

**Figure 4:**
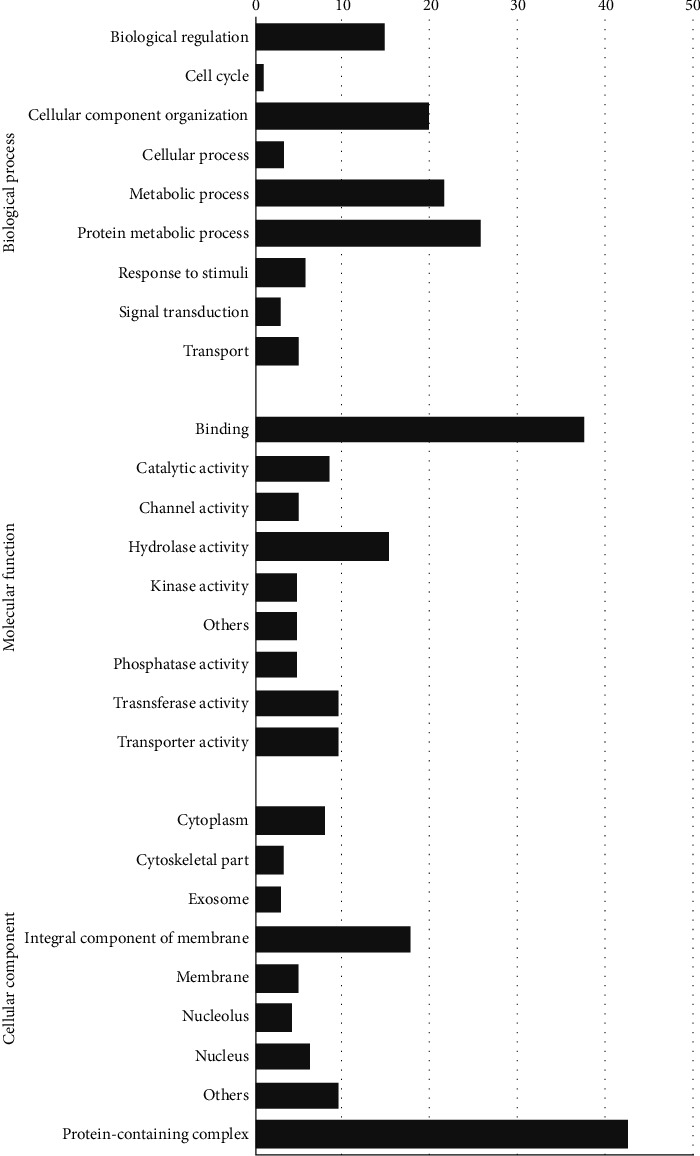
Percentage representation of GO enrichment analysis of lncRNA targets in *S. haematobium*.

In *S. haematobium*, protein metabolic processes (26%) and metabolic processes (21%) showed a consistently higher number of target genes. Also, the results indicate that the largest group of transcripts belongs to binding (38%) and protein-containing complexes (43%) related to molecular functions and cellular processes (Figure 4).

**Figure 5:**
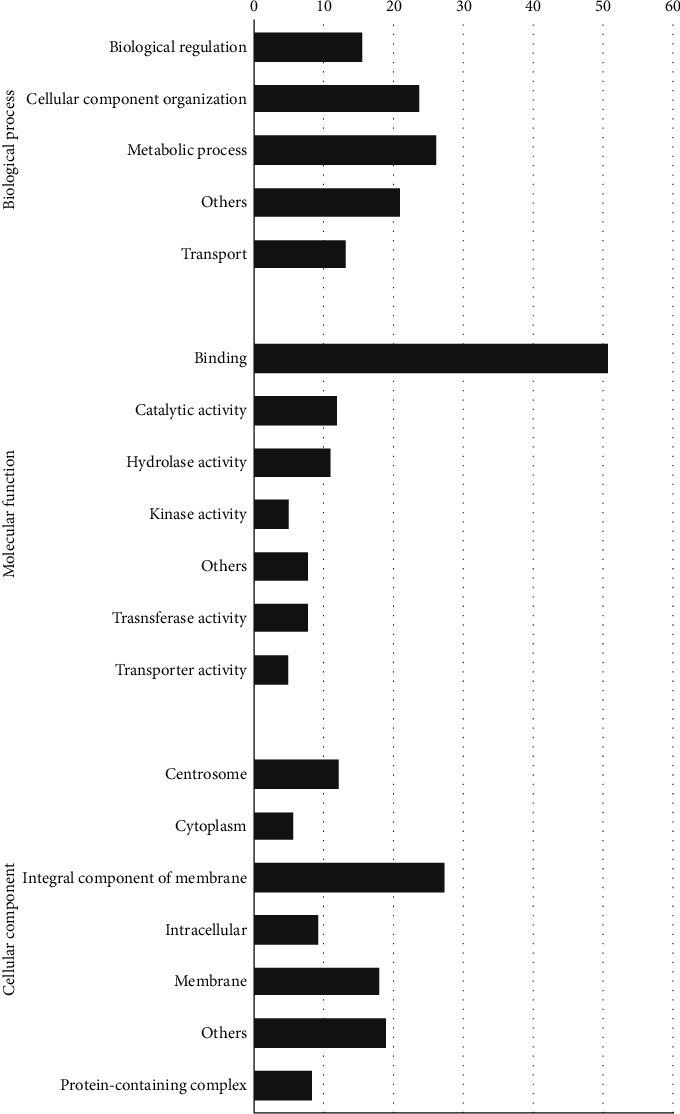
Percentage representation of GO enrichment analysis of shared lncRNA targets in *S. mansoni* and *S. haematobium*.

Among common target genes, the highest number of transcripts was involved in the metabolic process (26%) and cellular component organization (24%) in biological processes. The transcripts related to binding and integral membrane components had a significant number of target genes, respectively (Figure 5).

## 4. Discussion

Studying regulatory ncRNAs, such as miRNAs or lncRNAs, provides important clues about gene expression and/or evolutionary mechanisms. High-throughput next-generation sequencing has brought about the discovery of thousands of noncoding RNA genes (ncRNAs), such as short, long, and circular RNAs, over the last several decades. Also, several studies have been conducted for identification of both miRNAs and lncRNAs in *Schistosoma* genomes [25–27].

Schistosomiasis is a worldwide disease with insufficient data on its developmental processes and disease-inducing processes. Schistosomiasis is a worldwide disease with insufficient data on the molecular mechanisms of host-parasite interaction and the epigenetic changes involved in this process. Little is known about the genes and molecules that direct the biological process in the complex life cycle of the disease-causing parasite that includes different developmental stages. In this study, we aimed to determine the common homology-based lncRNAs between the two species using the comparative transcriptomic approach in both species adult male, female, and eggs, if there were any similar regulatory systems between these two species.

We detected 1669 and 899 lncRNAs of *S. mansoni* and *S. haematobium*. Besides, we found significant number of transcripts that were expressed in both *S. mansoni* and *S. haematobium* transcriptome libraries. In agreement with our findings, several studies indicate that a higher species similarity between *S. manson*i and *S. haematobium* in the case of lncRNAs and mRNAs exists [19]. Although we did not search the mRNA sequence similarity, BLASTN results give us a general insight about the lncRNA sequence similarity between these two species. Indeed, it has been reported that lncRNAs sequence conservation is lower than mRNA [17], and a reduction in levels of primary sequence conservation for lncRNAs among different organisms during evolution was found [13]. In this study, we detected a prominent number of similar lncRNAs between two species in egg, adult male, and adult female libraries. Previously, it was noticed that transcription of lncRNAs appears to be more active in the early embryonic stage [28]. This finding indicates that in early developmental stages, the same lncRNAs show parallel functions. In addition to a role in regulation of developmental gene activity, the role of lncRNAs was found to be widespread amongst metazoans [29, 30].

Different studies have revealed that lncRNAs play important roles in sexual development and determination in eukaryotes [31, 32]. In this study, a significant number of transcripts encoding lncRNAs located on chromosome 1 and sex chromosomes in *S. mansoni* were detected (Figure 1). In parallel, Oliveira et al. [26] reported in their research that the significant number of lncRNAs was found on chromosome 1, sexual ZW. It was indicated that there was a gene dosage differentiation in *Schistosoma* species [33]. Hence, we can argue about the functions of lncRNAs in sexual development.

Since lncRNA targets may consist of more than one target, they can influence gene expression in many biological processes [34]. In our study, some differences between *S. mansoni* and *S. haematobium* biological processes, molecular functions, and cellular compartments were found. A large number of target genes associated with the metabolic process have been identified in both *S. mansoni* and *S. haematobium*. On the other hand, while genes related to biological regulation were determined in large numbers in *S. mansoni*, genes related to protein metabolic function were found in large numbers in *S. haematobium*. In the study of Oliveira et al. [26], it was stated that most of the lncRNAs identified in *S. mansoni* were related to metabolism. As indicated before, these species were located in different tissues because their gene expression is regulated by different lncRNAs. Besides, parasite lncRNAs can play crucial roles in a tissue-specific manner in humans. It was shown by Maciel et al. [35] that some lncRNAs in *S. mansoni* have a tissue-specific expression in ovaries and testis. In this study, the authors obtained valuable information on the potential role of lncRNAs in schistosome reproduction. However, lncRNAs may be involved in a specific role related to the tissue in which they are located. Also, lncRNA gene expression can vary in different developmental stages in parasites; so, it is possible for these molecules to play an active role in the early diagnosis and treatment of the schistosomiasis. Although it is largely unknown whether lncRNA levels can be regulated by drugs, some studies have shown the effect of drugs on lncRNA expression [36–38]. A better understanding of lncRNAs and gene expression regulation mechanisms can help identify new therapeutic targets.

It has been reported that lncRNAs can be species-specific or even tissue-specific and that the sequence similarity organisms may be conserved in the evolutionary process [8]. Also, it was claimed that highly conserved sequences may be related to the functionality of lncRNAs among organisms [25]. However, we could not fully perform functional annotation for lncRNAs of schistosome due to minimal functional information of known genes and the limited number of RNA-seq samples. We found that *S. mansoni* and *S. haematobium* lncRNA sequences are conserved with 7.9% of the *S. haematobium* lncRNAs in alignment with the *S. mansoni* transcriptome.

## 5. Conclusions

In this study, we used the *S. mansoni* lncRNA library, so this process may explain why we detected more lncRNAs in *S. mansoni* than in *S. haematobium*. In addition, it is thought that lncRNAs, which are commonly expressed in accordance with the resulting data, are involved in both metabolic and biological regulation functions.

## Figures and Tables

**Table 1:** Long noncoding RNA transcripts showing high read numbers in *S. mansoni* and *S. haematobium*.

LncRNA	Read number	Target gene	Annotation
*S. mansoni*			
SmAS00026-IBu	27	Unidentified	Unidentified
SmLINC01664-IBu	24	Unidentified	Unidentified
SmAS00327-IBu	20	Smp_169460 (Rab, putative)	GTPase activity
SmLINC01664-IBu	16	Unidentified	Unidentified
SmAS00186-IBu	15	Uncharacterized protein	Unidentified

*S. haematobium*			
SmAS00186-Ibu	26	Uncharacterized protein	Unidentified
SmAS00167-Ibu	20	Unidentified	Unidentified
SmAS00326-Ibu	19	Unidentified	Unidentified
SmAS00328-Ibu	19	Unidentified	Unidentified
SmAS00041-Ibu	16	Unidentified	Unidentified
SmAS00042-Ibu	16	Smp_169460 (uncharacterized protein)	Cellular compound organization
SmAS00043-Ibu	16	Unidentified	Unidentified
SmAS00044-Ibu	16	Unidentified	Unidentified
SmAS00045-Ibu	16	Unidentified	Unidentified
SmAS00178-Ibu	16	Unidentified	Unidentified

## Data Availability

The data used to support the findings of this study are available from the corresponding author upon request.
